# Epicardial and Pericardial Fat Volume Correlate with the Severity of Coronary Artery Stenosis

**DOI:** 10.15171/jcvtr.2014.018

**Published:** 2014-12-30

**Authors:** Nasser Aslanabadi, Rezvanyeh Salehi, Alireza Javadrashid, Mohammadkazem Tarzamni, Behrouz Khodadad, Elgar Enamzadeh, Hossein Montazerghaem

**Affiliations:** ^1^Cardiovascular Research Center, Tabriz University of Medical Sciences, Tabriz, Iran; ^2^Department of Radiology, Tabriz University of Medical Sciences, Tabriz, Iran; ^3^Cardiovascular Research Center, Hormozgan University of Medical Sciences, Bandar Abbas, Iran

**Keywords:** Epicardial Fat Volume, Pericardial Fat Volume, Coronary Artery Disease, Multi Slice Computed Tomography

## Abstract

***Introduction:*** Epicardial fat volume (EFV) has been reported to correlate with the severity of coronary artery disease (CAD). Pericardial fat volume (PFV) has recently been reported to be strongly associated with CAD severity and presence. We aimed to investigate the relationship between EFV and PFV with severity of coronary artery stenosis in patients undergoing 64-slice multi-slice computed tomography (MSCT).

***Methods:*** One hundred and fifty one patients undergoing MSCT for suspected CAD were enrolled. Non-enhanced images were acquired to assess calcium score. Contrast enhanced images were used to quantify EFV, PFV and severity of luminal stenosis.

***Results:*** Coronary artery stenosis was mild in 25 cases (16.6%), moderate in 58 cases (38.4%) and severe in 68 cases (45%). With increase in severity of coronary artery stenosis, there was significant increase in PFV, EFV as well as epicardial fat thickness in right ventricle free wall in basal view and epicardial fat thickness in left ventricle posterior wall in mid and apical view. There was significant linear correlation between PFV with coronary calcification score (r=0.18, P=0.02), between coronary artery stenosis severity and PFV (r=0.75, P<0.001), EFV (r=0.79, P<0.001), apical epicardial fat thickness in right ventricle free wall (r=0.29, P<0.001), Mid (r=0.28, P<0.001) and basal (r=0.23, P=0.004) epicardial fat thickness in left ventricle posterior wall.

***Conclusion:*** PFV, EFV and regional epicardial thickness are correlated with severity of CAD and could be used as a reliable marker in predicting CAD severity.

## Introduction

Epicardial adipose tissue (EAT) is fat confined within pericardium. There is increasing evidence that regional visceral fat accumulation may contribute with unfavorable metabolic effects and cardiovascular risk factors.^1-3^ The adverse cardiovascular effects of obesity are mainly due to systemic and local accumulation of visceral fat pad with metabolically releasing of chemokines and cytokines.^4,5^

EAT as a fat depot is further implicated on coronary artery disease (CAD) because of proximity to the adventitia of major epicardial coronary arteries.^6,7^ EAT can be measured with simple echocardiography on free wall of right ventricle with correlation with presence of atherosclerotic CAD on conventional coronary angiography.^8^ Other imaging modalities for measurement of EAT are magnetic resonance imaging (MRI) and multi-slice computed tomography (MSCT).^9-11^ With recent developments in MRI and MSCT more comprehensive developments in measurements of EAT have become possible.^1^ Pericardial fat volume (PFV) is another marker that can be quantified from non-contrast computed tomography (CT) scans performed for coronary calcium scoring. PFV is strongly associated with CAD, coronary calcium score and severity of detected CAD.^12^

Unlike echocardiography, MSCT is capable of simultaneous demonstration of coronary calcium score, obstructive versus non-obstructive coronary lesions and also amount of EAT and epicardial fat volume (EFV). In this study we aim to evaluate the correlation between epicardial and PFV using MSCT with severity of the coronary artery stenosis.

## Materials and methods

### Study population

Between October 2011 and September 2012, a total of 151 adult patients who underwent MSCT with suspected CAD were included. Exclusion criteria were history of coronary bypass graft (CABG), CAD or allergy to contrast agents.

### CT imaging protocol

Prospective electrocardiographically triggered cardiac CT during single breath-hold using a dual-source computed tomographic system (Somatom Definition; Siemens Medical solution, Forchheim, Germany 64 slice scanner) at end–inspiration were used. Acquisition started above the origin of the coronary arteries and ended at the dome of the diaphragm. A tube voltage of 120 kV and tube current of 175 mAs for both tubes were used (dose –length product = 95 mGy/cm). Detector collimation was 1.2 mm and gantry rotation time was 0.33 ms. After manual adjustment of the field of view, data were reconstructed with 3 mm thickness with reconstruction increment of 1.5 mm and dedicated soft-tissue convolution kernel (b35F). All patients were in sinus rhythm at the time of study. For a patients with heart rate (HR) >60 beats/min, steady HR was achieved using β-blockers agents.

### Coronary artery calcium scoring

Atherosclerotic plaques were classified as calcified, non-calcified and mixed lesions. Each calcified segment of coronary arteries was scored by an experienced observer who blinded to clinical, CTA, and EAT volume analysis, using semiautomatic software available on the workstation. Presence of minimal three contiguous pixels with an attenuation of ≥ 130 Hounsfield unit (HU) was considered calcification; non-calcified were defined as structures clearly assignable to the vessel wall (in at least two views) with density lower than the lumen contrast; Plaques demonstrating calcification ≤50% of the plaque area were classified as mixed. Coronary artery calcification score were calculated using the method described by Agatston et al.^13^

### Plaque evaluation

Optimal data set was chosen after evaluation of all reconstructed data sets at different ECG–phases. The MSCT datasets were analyzed by two independent investigators blinded to EFV and clinical characteristics of patients. No CAD was defined as no visible coronary atherosclerotic plaques; mild CAD (non-obstructive) was considered plaques causing <50% luminal narrowing, Moderate CAD as plaques causing 50-70% luminal narrowing and severe as plaques causing >70% luminal narrowing.

### Pericardial and epicardial adipose tissue volume measurements

Pericardial fat was defined as adipose tissue enclosed by the visceral pericardium, including fat directly surrounding the coronary arteries. For defining pericardial contours, the upper slice limit, marked by bifurcation of the pulmonary trunk, and lower slice limit, identified as the slice just below the posterior descending artery, were chosen. This lower limit was chosen to better distinguish pericardial fat from fat around the diaphragm. Epicardial fat was identified on contrast-enhanced CT as a hypodense rim around the myocardium and limited by pericardium. Quantification of total fat volume was done, as described by Gorter et al.^14^ The visceral pericardium, was traced manually from the mid left atrium to the left ventricular apex, and all extra-pericardial tissue was excluded. Then the images were segmented using an attenuation threshold varying between -250 HU and -30 HU providing the EAT area in each slice. With this method coronary arteries and calcium, myocardium, the aorta and blood pool, effectively were excluded. The EAT area at each level was summed across slices and multiplied by the slice thickness and number of all slices to determined total EFV. The reproducibility of the method was described by the Gorter et al.^14^ and Nichols et al.^15^

### Statistical analysis

All data were analyzed using the Statistical Package for Social Sciences, version 17.0 (SPSS, Chicago, Illinois). Baseline data are reported as means ± standard deviation (continuous data) or percentages (categorical data), depending on the data level. In order to analyze the differences between the groups in the quantitative variables, Student’s *t*-test was used in those with normal distribution and the Mann–Whitney U test if the distribution was not normal. The association between qualitative variables was studied by means of Chi-square test or Fisher’s exact test. Pearson’s correlation coefficient was calculated to assess the relation between different variables. A *P* value <0.05 was considered significant.

## Results

One hundred and fifty one patients were enrolled in this study. Patients’ baseline findings are shown in Table 1. Coronary artery stenosis was mild in 25 cases (16.6%), moderate in 58 cases (38.4%) and severe in 68 cases (45%). Measurement of EFV was feasible in all patients. Mean PFV was 87.74±34.69 ml and mean EFV was 72.09±35.50 ml. There were 59 cases with diagnosed 1 vessel, 2 vessel or 3 vessel coronary disease. LAD was present in all cases alone or with other coronary arteries.

**Table 1:** Patients’ baseline findings

	**Number (%)/Mean±SD**
Age (years)	53.22±11.23
Gender	
Male	86 (57%)
Female	65 (43%)
Hyperlipidemia	46 (30.5%)
Diabetes mellitus	25 (16.6%)
Hypertension	61 (40.4%)
Smoking	23 (15.2%)
Familial History	47 (31.1%)
Left main coronary artery	9 (6%)
Left anterior descending artery	59 (39.1%)
Left circumflex artery	29 (19.2%)
Right coronary artery	29 (19.2%)

Table 2 demonstrates epicardial and PFV and epicardial fat thickness in different severity of coronary artery stenosis. There was significant difference between different severity of the coronary stenosis in PFV, EFV as well as epicardial fat thickness in right ventricle free wall in basal view and epicardial fat thickness in left ventricle posterior wall in mid and apical view, which all significantly increase by increase in the stenosis severity.

**Table 2:** Epicardial and pericardial fat volume and epicardial fat thickness in different severity of coronary artery stenosis

	**Severity of coronary artery stenosis**			***P *** **value**
	**Mild**	**Moderate**	**Severe**	
Pericardial fat volume	49.06±17.81	72.05±14.53	115.78±28.97	<0.001
Epicardial fat volume	29.93±9.37	54.98±8.80	102.21±29.80	<0.001
Basal epicardial fat thickness in right ventricle free wall	0.45±0.19	0.54±0.22	0.63±0.19	0.001
Mid epicardial fat thickness in right ventricle free wall	0.51±0.31	0.60±0.25	0.61±0.19	0.21
Apical epicardial fat thickness in right ventricle free wall	0.61±0.35	0.62±0.23	0.65±0.27	0.68
Basal epicardial fat thickness in left ventricle posterior wall	0.57±0.28	0.56±0.29	0.57±0.20	0.98
Mid epicardial fat thickness in left ventricle posterior wall	0.36±0.24	0.44±0.15	0.50±0.16	0.002
Apical epicardial fat thickness in left ventricle posterior wall	0.46±0.19	0.56±0.23	0.51±0.21	0.01

Correlations between CAD risk factors and EFV and PFV were evaluated and found negative correlation between PFV with smoking (r=-0.17, P=0.03) and positive correlation with age (r=0.27, P=0.001). There was also positive correlation between age and EFV (r=0.24, P=0.002). There was significant linear correlation between PFV with coronary calcification score (r=0.18, P=0.02), but the correlation between EFV and coronary calcification score was not significant (r=0.14, P=0.08).

There was also significant linear correlation between coronary artery stenosis severity and PFV, EFV, apical epicardial fat thickness in right ventricle free wall, Mid and basal epicardial fat thickness in left ventricle posterior wall (Table 3).

**Table 3:** Pearson’s correlation coefficients between coronary artery stenosis severity and regional epicardial fat thickness and epicardial and pericardial fat volume

**Correlation of coronary artery stenosis severity with:**	**Pearson’s correlation coefficient**	***P*** ** value**
Pericardial fat volume	0.75	<0.001
Epicardial fat volume	0.79	<0.001
Apical epicardial fat thickness in right ventricle free wall	0.29	<0.001
Mid epicardial fat thickness in right ventricle free wall	0.12	0.11
Basal epicardial fat thickness in right ventricle free wall	0.07	0.39
Apical epicardial fat thickness in left ventricle posterior wall	-0.003	0.97
Mid epicardial fat thickness in left ventricle posterior wall	0.28	<0.001
Basal epicardial fat thickness in left ventricle posterior wall	0.23	0.004

## Discussion

Because MSCT is increasingly used as a clinical and research tool, measurement of EFV may provide additional information about risk stratification.^16-18^ In this study EFV was accurately determined by dual source 64 slice CT and the severity of stenotic coronary arteries were determined by coronary CT angiography. Our results showed that EFV and PFV had positive correlation with age among traditional cardiovascular risk factors and also there was negative correlation between PFV and smoking. We present a good relation between PFV and EVF with presence of CAD. Our findings are inconsistence with recent population based studies that convincingly linked EFV to cardiovascular events.^15-17^ Also, previous studies with MSCT revealed that EFV was higher in patients with CAD as compared with patients without CAD.^19,20^ It is also shown that pericardial fat is correlated with cardiovascular disease risk factors.^21^

Previous studies have shown strong correlation between pericardial fat and coronary artery calcium score as well as cardiac events. It is recommended that pericardial fat has a direct role in coronary atherosclerosis.^22-25^ The association between EFV and coronary calcification is also shown in previous studies.^21,26^ Similarly, we found significant correlation between PFV with coronary calcification score, but the correlation between EFV and coronary calcification score was not significant.

EAT accounts for about 20% of total heart weight and equally distributed on surface of the right and left ventricle.^27^ The most prominent physiologic function of EAT are first protective effect of coronary arteries against torsion induced by arterial pulsation and cardiac contraction as well as protective effect on coronary arteries against trauma.^1^ Second, it serves as a buffering system against toxic effects of high levels of circulatory fatty acids (FFA) by its ability to scavenge excess fatty acids. Third, the increased lipolytic activity of EAT suggests that this fat depot may serve as a local energy source by producing of FFAs under high metabolic demands during ischemia.^28^ Forth is the protective role for cardiac ganglia.^29^ On the other hand EAT could been released a significant amount of inflammatory mediators linked to cardiovascular disease.^9,30^ The presence of this substances in direct proximity of coronary arteries may influence inflammatory responses and also coronary calcification at late stages.^30^

Epicardial fat thickness reflects visceral adiposity rather than general obesity. It correlates with metabolic syndrome, insulin resistance, CAD, and subclinical atherosclerosis, and could serve as a simple tool for cardiometabolic risk prediction.^31^ We also find significant correlation between coronary artery stenosis severity and PFV, EFV, apical epicardial fat thickness in right ventricle free wall, Mid and basal epicardial fat thickness in left ventricle posterior wall. Similarly Rajani et al.^32^ showed significant association between EFV and severe coronary stenosis. Wang et al.^29^ also showed that epicardial fat thickness was correlated to the extent and severity of significant CAD.

## Limitations

There are some limitations in our study. First, this is a cross-sectional study and shows only association and so the cause and outcome was not evaluated. Second, markers of inflammation were not determined in our study, and these markers may provide additional support for causal association between EFV and CAD. Third, some degree of ethnic differences may be present about EFV. We did not review intra-abdominal visceral fat which is closely related to adverse cardiovascular events. Radiation hazards are another limitation of study. Also EFV and PFV were not connected for BMI in our study.

## Conclusion

PFV and EFV and regional epicardial thickness are correlated with severity of CAD and could be used as a reliable marker in predicting CAD severity.

## Acknowledgments

This article was written based on a dataset of cardiology residency thesis, registered in Tabriz University of Medical Sciences.

## Ethical issues

The study protocol was approved by the ethics committee of Tabriz University of Medical Sciences and all subjects gave written informed consent.

## Competing interests

Authors declare no conflict of interest in this study.
